# Attributes of *Anopheles gambiae* swarms in South Central Uganda

**DOI:** 10.1186/s13071-024-06132-9

**Published:** 2024-03-21

**Authors:** Krystal Birungi, Danspaid P. Mabuka, Victor Balyesima, Frederic Tripet, Jonathan K. Kayondo

**Affiliations:** 1 Entomology Division, Uganda Virus Research Institute (UVRI), Plot 51-59, P.O. Box 49, Entebbe, Uganda; 2 Swiss Tropical and Public Health Institute, Kreuzgasse 2, 4123 Allschwil, Switzerland

**Keywords:** Swarm, Mixed swarms, Mating behaviour, *Anopheles gambiae*, Vector ecology, Sampling, Malaria

## Abstract

**Background:**

*Anopheles gambiae* continues to be widespread and an important malaria vector species complex in Uganda. New approaches to malaria vector control are being explored including population suppression through swarm reductions and genetic modification involving gene drives. Designing and evaluating these new interventions require good understanding of the biology of the target vectors. *Anopheles* mosquito swarms have historically been hard to locate in Uganda and therefore have remained poorly characterized. In this study we sought to identify and characterize *An. gambiae* s.l mosquito swarms in three study sites of high *An. gambiae* s.l prevalence within Central Uganda.

**Methods:**

Nine sampling visits were made to three villages over a 2-year period. Sampling targeted both wet and dry seasons and was done for 2 days per village during each trip, using sweep nets. All swarm data were analysed using the JMP 14 software (SAS Institute, Inc., Cary, NC, USA), parametrically or non-parametrically as appropriate.

**Results:**

Most of the *An. gambiae* s.s. swarms sampled during this study were single-species swarms. However, some mixed *An. gambiae* s.s. and *Culex* spp. mosquito swarms were also observed. Swarms were larger in the wet season than in the dry season. Mean swarm height ranged from 2.16 m to 3.13 m off the ground and only varied between villages but not by season. *Anopheles gambiae* mosquitoes were present in all three villages, preferred to swarm over bare ground markers, and could be effectively sampled by field samplers.

**Conclusions:**

This study demonstrated that *An. gambiae* s.l swarms could be effectively located and sampled in South Central Uganda and provided in-depth descriptions of hitherto poorly understood aspects of *An. gambiae* local swarm characteristics. Swarms were found close to inhabited households and were greater in size and number during the rainy season. *Anopheles gambiae* s.s swarms were significantly associated with bare ground markers and were sometimes at heights over 4 m above the ground, showing a necessity to develop tools suitable for swarm sampling at these heights. While mixed species swarms have been reported before elsewhere, this is the first documented instance of mixed genus swarms found in Uganda and should be studied further as it could have implications for swarm sampling explorations where multiple species of mosquitoes exist.

**Graphical Abstract:**

**Figure:**
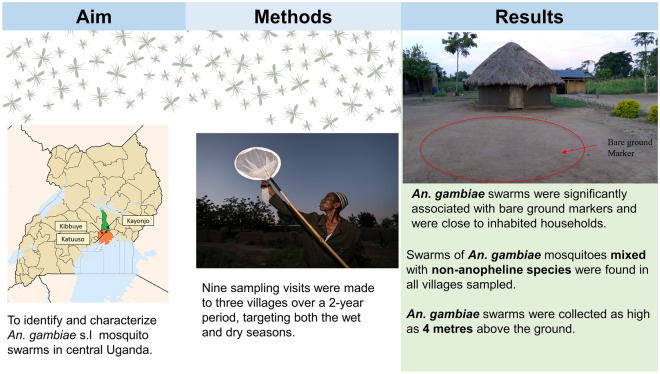

**Supplementary Information:**

The online version contains supplementary material available at 10.1186/s13071-024-06132-9.

## Background

In 2021, there were an estimated 247 million malaria cases worldwide, with four African countries accounting for almost 50% of the total malaria infections. Among these, Uganda carried the third highest malaria case burden [1].

In Africa, sibling mosquito species belonging to the *Anopheles gambiae* species complex are some of the most widespread and important malaria vectors. *Anopheles coluzzii* and *An. gambiae* s.s. have historically been implicated in malaria transmission in many areas because of their high levels of endophily and anthropophilly. These factors, combined with these species' ability to continuously adapt to interventions against them, explain why *An. gambiae* s.l is one of the most important malaria vector species complexes in the world [2]. A number of new approaches for malaria vector control are being explored including population suppression through swarm reductions [3] and genetic modification involving gene drives [4–6]. However, development and evaluation of these new interventions will require an understanding of the biology of target vectors and of their mating behaviour in particular.

Swarming is an integral part of mating in the Anophelines. Males congregate at dusk over contrasting markers on the ground and female mosquitoes visit these swarms to select a mate and engage in copula. Male *An. gambiae* s.l mosquitoes have been found to congregate at the same locations regularly every day [3, 7, 8]. This makes swarm sampling an ideal method for the study of male mosquitoes and mosquito mating behaviour. It also makes the identification and targeting of *An. gambiae* s.l mosquito swarms a largely unexplored avenue for possible mosquito control [3, 9]. If swarms can reliably be identified, then there is a possibility that male mosquitoes can be targeted for mosquito population control in that location as has previously been demonstrated on a small scale in West Africa [3]. Anopheles mosquito swarms have historically been hard to locate in Uganda. To our knowledge, until 2017, the last published report on swarms in East Africa as a whole dated back from 1980 [10]. Anopheles swarms have recently been identified and characterised in studies from Tanzania [11, 12]. However, they focused on *Anopheles arabiensis* and *An. funestus* and not *An. gambiae* s.s., which is also an important species and more prevalent in Uganda and many other areas in nearby countries [2, 13–15]. A study of male mosquito collection methods in Uganda did recently report on Anopheles swarm location but did not do much characterization of the swarms themselves [16]. Far more publications are available on mosquito swarms from West Africa where the co-occurrence of *An. gambiae* s.s., *An. coluzzii*, and *An. arabiensis* has been a springboard for studies focusing on speciation and assortative mating [17, 18]. Given the importance of understanding mating behaviour for innovative approaches including mosquito release strategies and swarm killing approaches, combined with the paucity of data on swarm dynamics originating from East Africa, this study aimed to identify and characterize *An. gambiae* s.l mosquito swarms in three study sites of high *An. gambiae* s.l prevalence within Central Uganda, thereby addressing an important knowledge gap in the reproductive biology of this key malaria vector species.

## Methods

### Study area

**Fig. 1:**
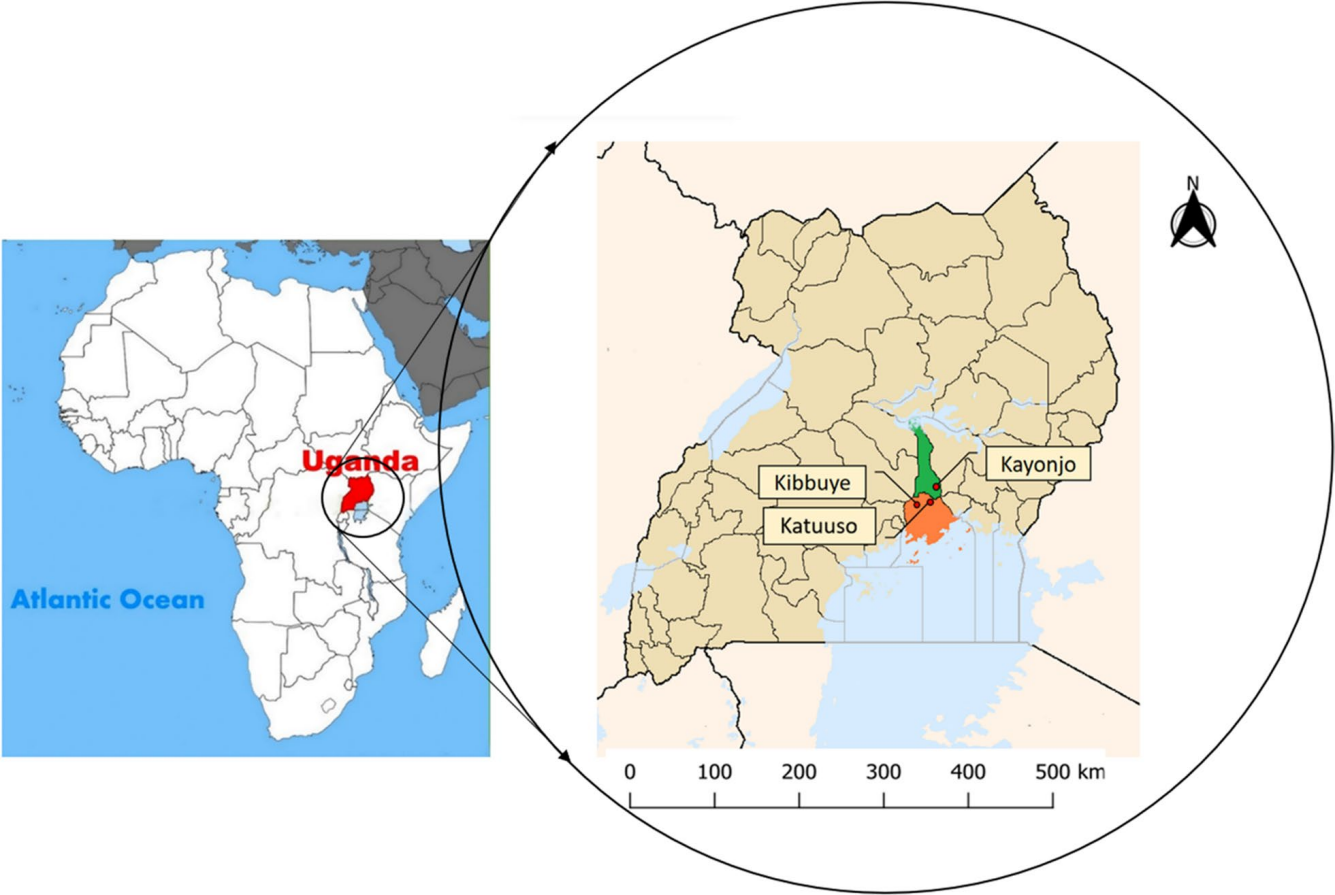
Location of the study sites in Uganda. The study villages of Kibbuye (KY) and Katuuso (KT) are in Mukono district and Kayonjo (KJ) is in Kayunga district. All are approximately 50 km NW of the capital city, Kampala

The focus of this study was the location and characterisation of *An. gambiae* s.s swarms in three village study sites in south central Uganda. The parameters studied included swarm species composition, height above ground, swarm sizes, swarm marker preference, and swarm distribution within the villages. Swarm sampling was done at Kibbuye (KY) and Katuuso (KT) villages in Mukono district and Kayonjo (KJ) village in Kayunga district (Fig. 1) in 2017 and 2018. These villages typically experience two rainy seasons and two dry seasons per year. The first rainy season is generally from March to June, followed by a dry season during July to August. The second rainy season runs from September to November and is followed by a dry period from December to February. All three sites record high malaria incidence (up to 150 confirmed malaria cases per 1000 population/year) and are located in areas of high malaria endemicity [2]. In this region *An. gambiae* s.s is the dominant species of the *An. gambiae* complex and the vector responsible for most of the malaria [2].

The village of Kibbuye is located in Seeta-namuganga sub-county of Mukono district (0.724N, 32.784E) and has approximately 1500 inhabitants. The major economic activity is agriculture and most gardens are located in the swampy village borders where village residents grow rice. Coffee plants are also grown more centrally within the village and make up most of the bushy vegetation present within the village. Cows, pigs, and goats are also kept by families on a small scale (< 10 animals per household). Most mosquito larval habitats are found in the rice gardens which make use of naturally occurring ground water and therefore provide relatively permanent breeding sites, although these pools can still dry out in periods of extended drought. Mosquito larval habitats are also found in local rock pools of collected rainwater. These are only semi-permanent because they dry up quickly when there is no rainfall.

Katuuso village is also located in Seeta-namuganga and seven kilometres southeast of Kibbuye (0.699N, 32.843E); Katuuso has approximately 800 inhabitants. The major economic activity is agriculture, and the gardens are located all around the swampy village borders. Residents in Katuuso village grow rice as well as various annual food crops such as sweet potatoes and maize. Animal husbandry is also present on a small scale where families keep cows, pigs, and goats, with fewer than five animals per home. Coffee plants are present throughout the village although not on as large a scale as in Kibbuye. The mosquito larval habits are located in the crop gardens, which can be found mainly around the swampy village borders. These pools are also permanent aside from cases where a long drought dries up the swamp water.

Kayonjo village is located in Busaana sub-county of Kayunga district (0.925N, 32.862E) and has approximately 1800 inhabitants. Located approximately 10 km east of the other two villages, it is similar to them in that the major economic activity is agriculture with large tracts of swampy ground at the village borders where most for the farming takes place. A diversity of food crops is grown in Kayonjo village including maize, sweet potatoes, rice, and yams. Vegetation within the village is composed of large evergreen trees as well as many coffee bushes throughout the village. The most abundant trees are mango trees along with a few other fruit trees. Livestock farming also present in Kayonjo village on a very small scale with some residents owning one or two cows. Mosquito larval habitats in Kayonjo village are found mostly in the swampy gardens on the village borders and are also permanent sites provided there is no prolonged drought.

### Swarm sampler training

UVRI entomology field staff underwent a training in May 2015 conducted by a team from the Institut de Recherche en Sciences de la Santé (IRSS) located in Burkina Faso. IRSS has pioneered mating studies based on Anopheline swarm sampling and have published a number of seminal publications on the subject [23, 23, 23]. Over a 7-day period, UVRI field staff members were trained in the following techniques: (1) swarm marker identification and location methods; (2) swarm identification and characterisation; (3) swarm sampling, sample processing, and storage for later molecular identification.

Surveys were carried out in 2016 to locate mainland field sites for the study of *An. gambiae* mosquito populations as part of a larger project within the Department of Entomology at the Uganda Virus Research Institute. Among the criteria for site selection was the presence of *An. gambiae* species of mosquitoes; these sites were therefore ideal for this swarm study. Swarm surveys commenced at selected sites in 2017. Prior to swarm surveys, UVRI entomologists then trained local volunteers consisting of adult males ≥ 18 years of age in swarm sampling techniques including swarm marker identification and location, swarm characterisation, and swarm sampling using sweep nets. While biting risk was low since swarm sampling targets male mosquitoes, swarm sampling volunteers were still educated on malaria and mosquitoes and how to dress to reduce the chances of mosquito bites (long-sleeved shirts and trousers). Samplers were also taught the purpose of the swarm sampling exercise and gave their verbal consent before the start of each sampling activity.

### Sampling approach

**Fig. 2:**
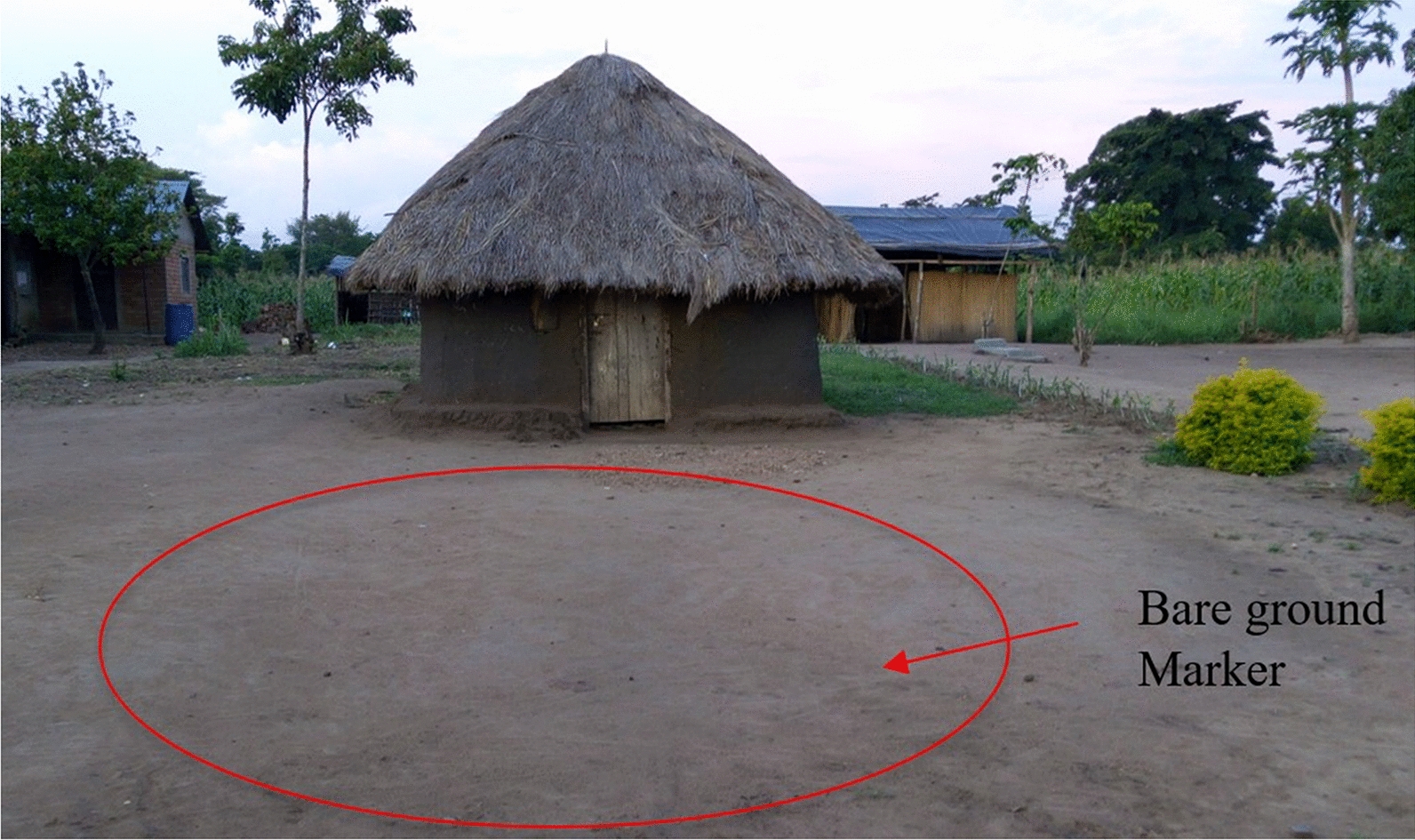
An example of a bare ground marker above which mosquito swarms were observed and sampled within the villages

Nine sampling visits were made over a 2-year period. Sampling targeted both wet and dry seasons to capture swarm dynamics throughout the year. The wet season sampling took place in March 2017, May 2017, October 2017, November 2017, April 2018, May 2018, and September 2018; the dry season sampling was done in December 2017 and July 2018. The study sites were sampled sequentially during each trip, starting with Kibbuye village and ending with Kayonjo village. Each village was sampled for 2 days during these trips. Each village was notionally divided into two halves and mosquitoes were sampled from each half during the 2 sequential days of each survey. Swarm sampling was done following the method described by Diabate et al. [18]. Twenty-eight swarm samplers worked in pairs, locating points of contrast (markers) on the ground, for example bare ground surrounded by grass or vice versa, sand heaps, tarpaulins, etc., throughout the village (Fig. 2). This activity started at 18:00 on each day, where samplers located these markers and then watched the spaces above these markers against the lighter background of the sky at dusk. When a swarm was seen, the samplers made two sweeps of the swarm using a sweep net. The sweep net was then tied off with a knot leaving the sampled mosquitoes at the bottom of the net. Swarm surveys continued until the end of the swarming period at complete sunset. Coordinates of each swarm seen were taken using a portable handheld Garmin GPS. The swarm was given a location code on a data sheet. The swarm size in numbers of mosquitoes and the height from the bottom of the swarm to the ground was estimated by eye by the swarm sampler and noted on the data sheet. The marker associated with the swarm was also recorded along with the date and the name of the village. Each net with a sample was labelled with the location code and stored overnight to wait for identification in broad daylight to avoid the risk of misidentification and escape in the dark nighttime conditions after swarm sampling was complete. The following morning, mosquitoes were removed from the nets and killed with chloroform for morphological identification. All mosquitoes sampled were morphologically identified to species in the field in broad daylight by a trained entomologist equipped with a field microscope and a morphological key [19, 20]. The species and sex of the mosquitoes sampled were recorded. Morphologically identified *An. gambiae* s.l samples were then placed in a clearly labelled 1.5-ml tube and stored in 80% ethanol for transport to the laboratory at UVRI for additional molecular confirmation. Up to 20 mosquitoes were stored per tube, provided they originated from the same swarm.

### Mosquito molecular identification

Mosquito molecular identification was done using a polymerase chain reaction (PCR) species identification protocol described by Wilkins et al. [21]. The PCR cycling was done on a PTC-100 thermocycler (MJ Research Inc, Watertown, MA, USA) and consisted of melting at 95 °C for 5 min followed by 30 cycles of 95 °C for 30 s, 58 °C for 30 s, and 72 °C for 30 s, followed by one cycle of 72 °C for 5 min. Each reaction comprised template DNA (2 ng) from a single mosquito, primers (1 µM), MgCl_2_ (0.3 mM), dNTPs (0.08 mM), Taq polymerase (1U) (Invitrogen, Life Technologies corp. Carlsbad, CA, USA), Go green Taq buffer (1x) (Invitrogen, Life Technologies corp. Carlsbad, CA, USA), and dH_2_0 topped to 25 μl total reaction volume. PCR products (10 ul) were observed by separation on Agarose (1%) TBE gels stained with ethidium bromide and run in 0.5 × TBE buffer at 12 v/cm for 25 min after which the gels were visualized by ultraviolet illumination and fragment sizes were estimated using a 1-kb ladder marker. The primers used were IMP-UN (5’-GCTGCGAGTTGTAGAGATGCG-3’) as a forward primer and QD-3 T (5’-GCATGTCCACCAACGTAAAATCC-3’), ME-3 T (5’-CAACCCACTCCCTTGACGATG-3’), GA-3 T (5’-GCTTACTGGTTTGGTGCGGCATGT-3’), and AR-3 T (5’-GTGTTAAGTGTCCTTCTCCGTC-3’) reverse primers.

### Statistical analysis

All swarm data were analysed using JMP 14 software (SAS Institute, Inc., Cary, NC, USA) [22]. All data were checked for normality and subsequently analysed parametrically or non-parametrically as appropriate. General linear models were used to detect significant variations in relation to the parameters tested and non-significant interactions were removed in a step-wise manner. All linear models were checked for heteroscedasticity and outliers.

## Results

### Swarm distribution within the villages

**Fig. 3:**
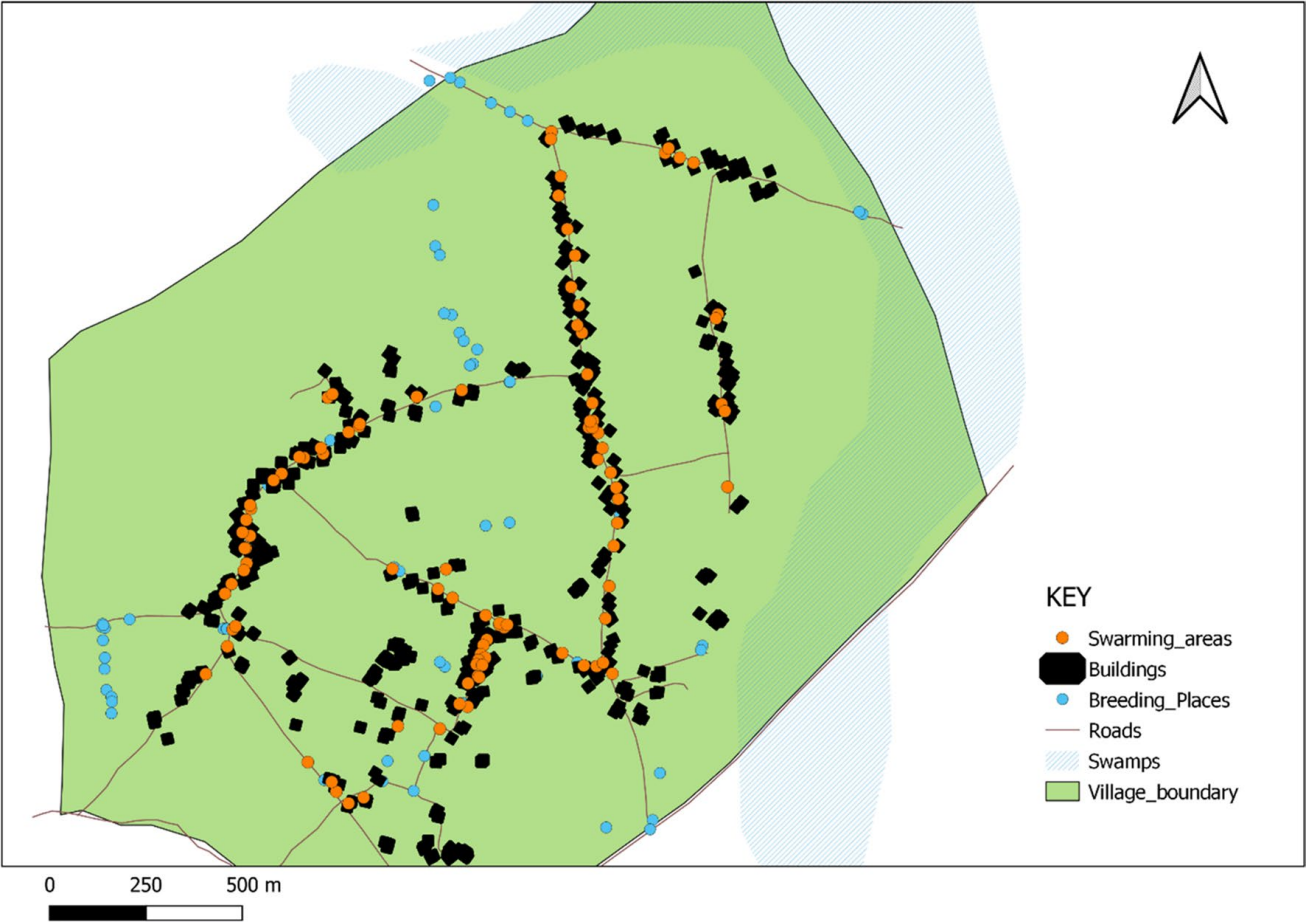
Map of Kayonjo village showing the distribution of mosquito swarms

**Fig. 4:**
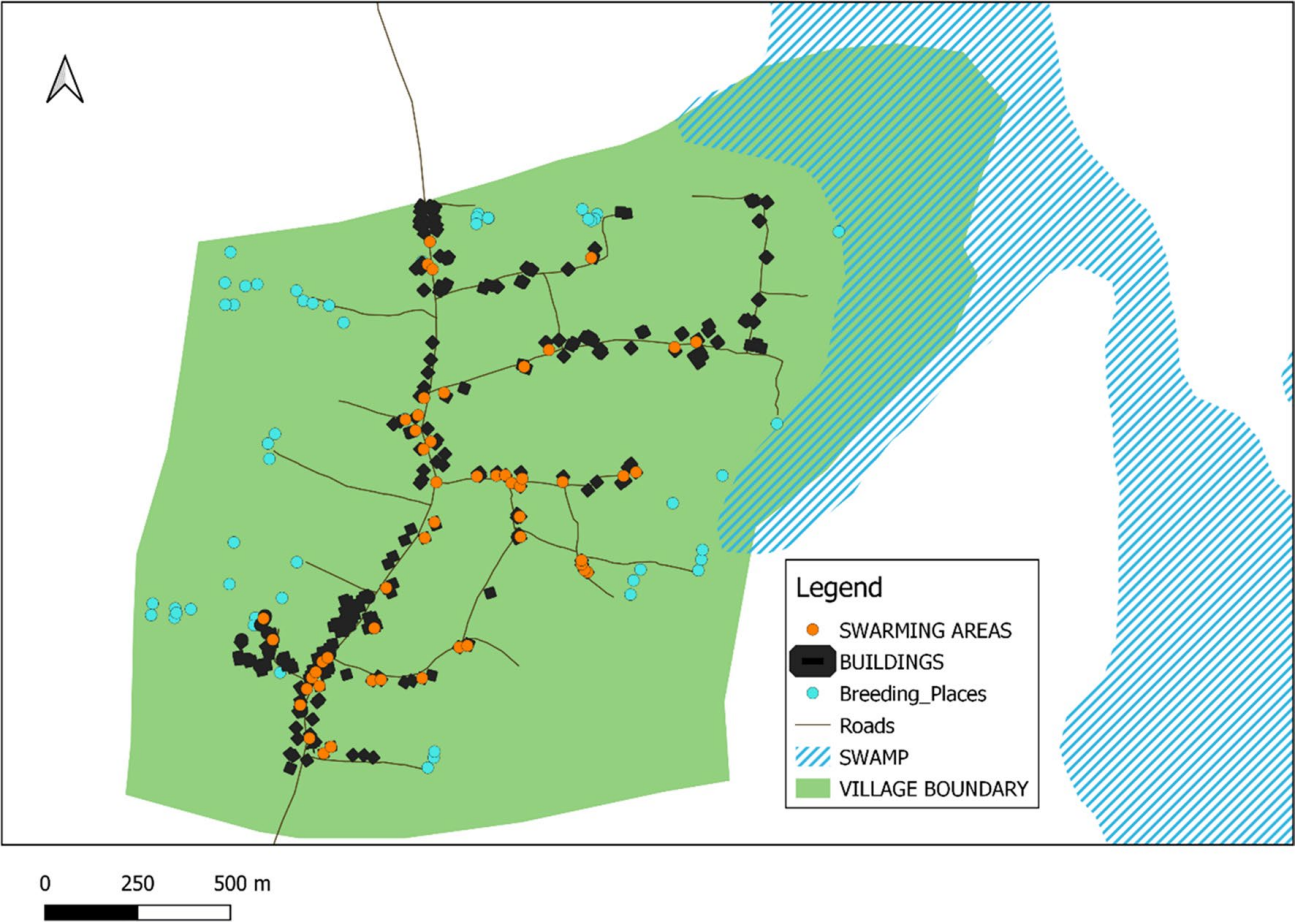
Map of Katuuso village showing the distribution of mosquito swarms

*Anopheles gambiae* swarms were sampled throughout the inhabited areas of all three villages. Houses in all three villages are situated along village paths. Swarms were located mainly inside household compounds within 1 to 10 m of buildings, in contrast to larval habitats which were mostly located along village boundaries (Figs. 3, 4).

### Swarm characteristics

**Fig. 5:**
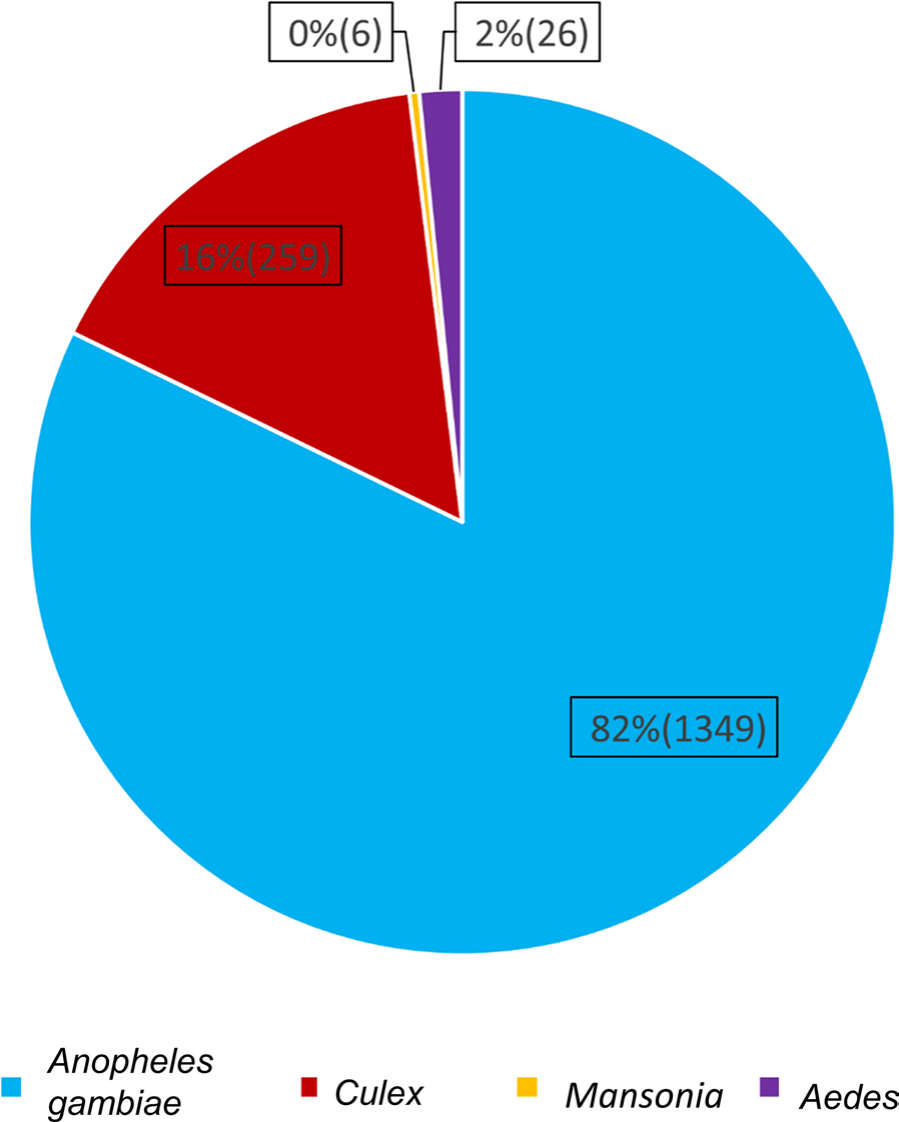
Percentage (*N*) of each mosquito species captured out of the total sampling over the three villages

**Fig. 6:**
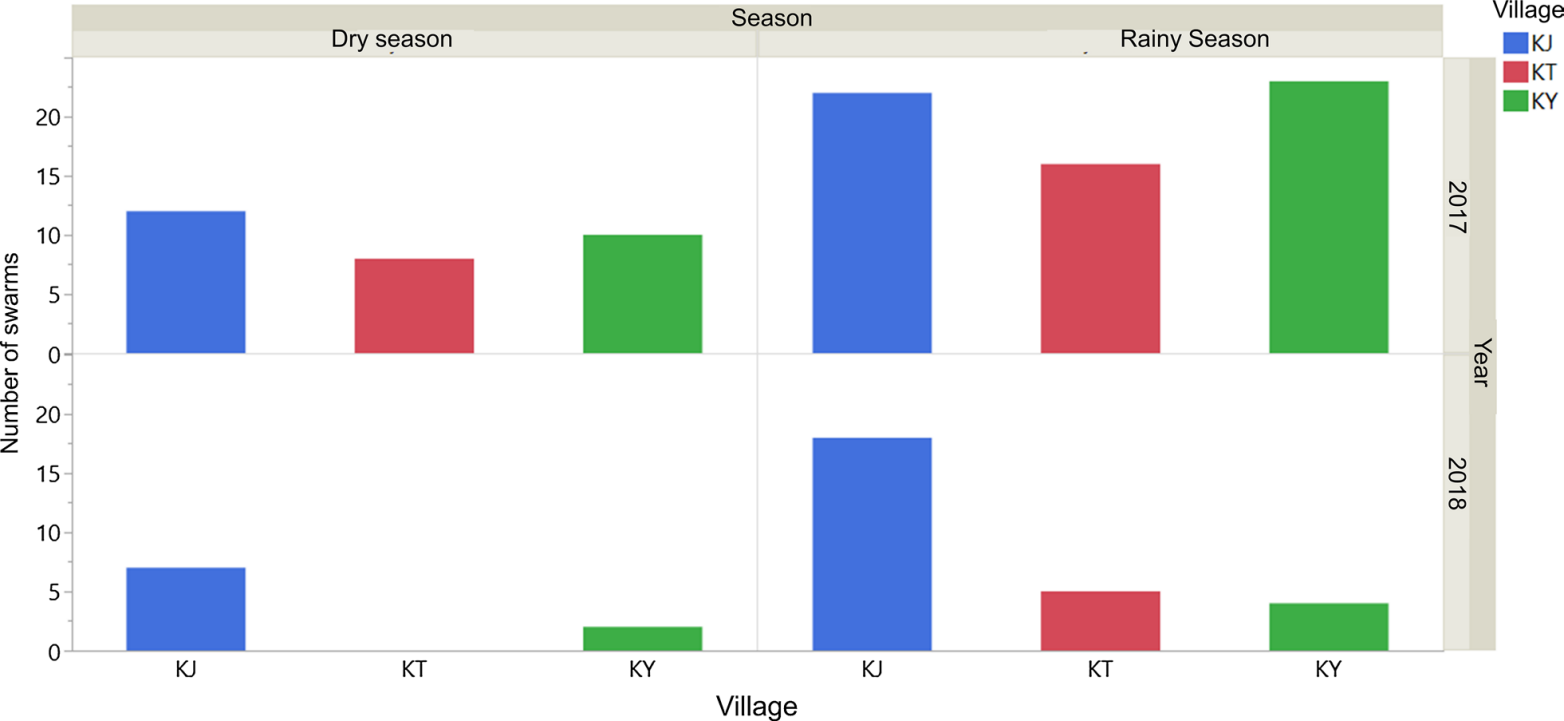
Swarm abundance by season and village

Mosquito swarms were observed in all three of the villages. A total of 127 mosquito swarms were located and sampled during this study, and a total of 1636 mosquitoes were sampled from them. Of these, 82% (*n* = 1345) were *An. gambiae* s.l., 16% (*n* = 256) were *Culex* spp., 2% (*n* = 86) were *Aedes* spp., and < 1% (*n* = 6) were *Mansonia* spp. (Fig. 5). More mosquito swarms were recorded during the rainy seasons compared to the dry seasons in all three villages regardless of the sampling year (Poisson distribution general linear model: *df* = 3, likelihood ratio LR = 30.2, *P* < 0.001) (Fig. 6).

### Swarm sizes

**Fig. 7:**
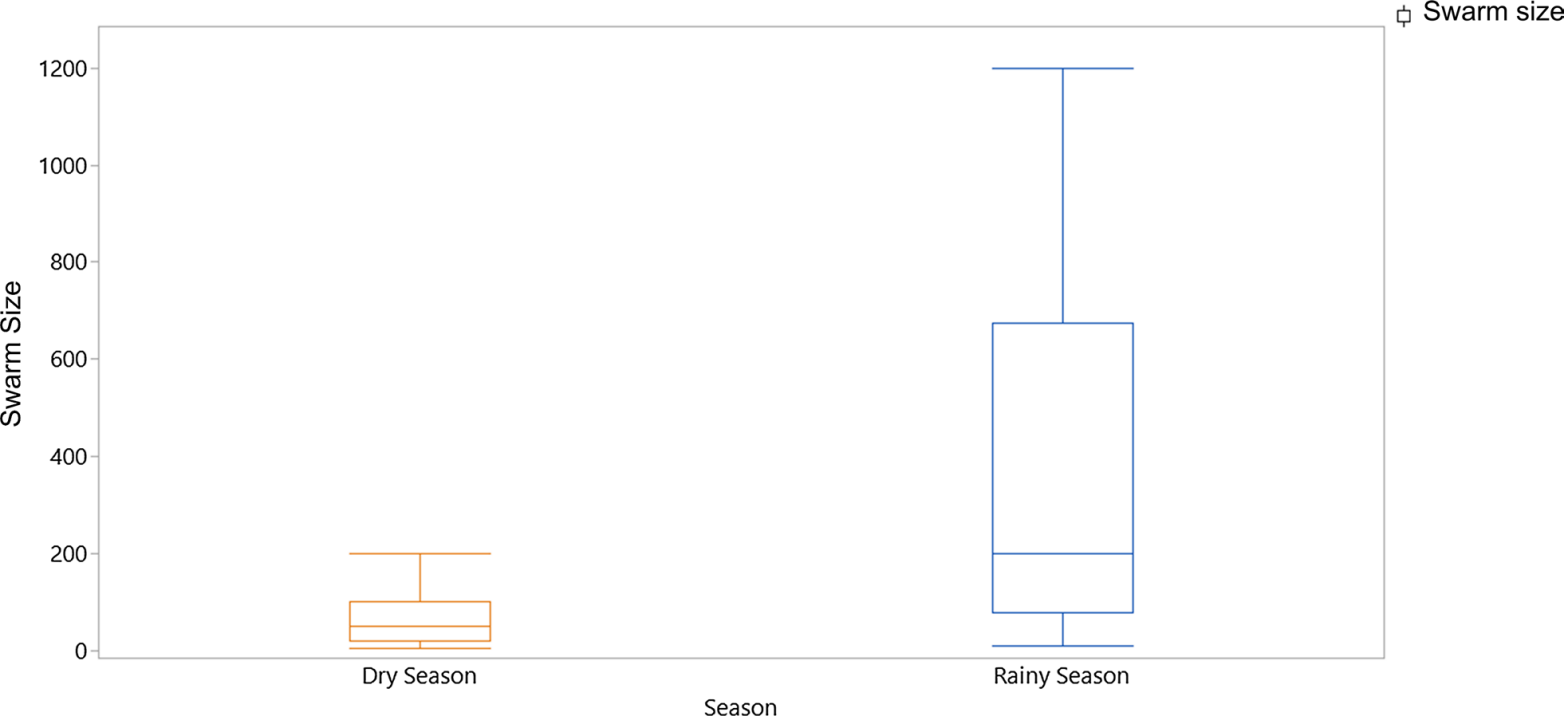
Variation in median swarm size by season

There was no difference in the swarm size between the villages but swarm sizes varied significantly between the wet and dry season (Fig. 7), with the dry season having smaller swarms than was observed during the rainy season (Poisson distribution general linear model: village: *df* = 2, likelihood ratio LR = 0.7, *P* = 0.701; season: *df* = 1, LR = 9.9, *P* < 0.002). Swarm sizes did not correlate significantly with swarm height (Spearman’s rank correlation: *r* = 0.051, *P* > 0.05).

### Swarm species composition

**Fig. 8:**
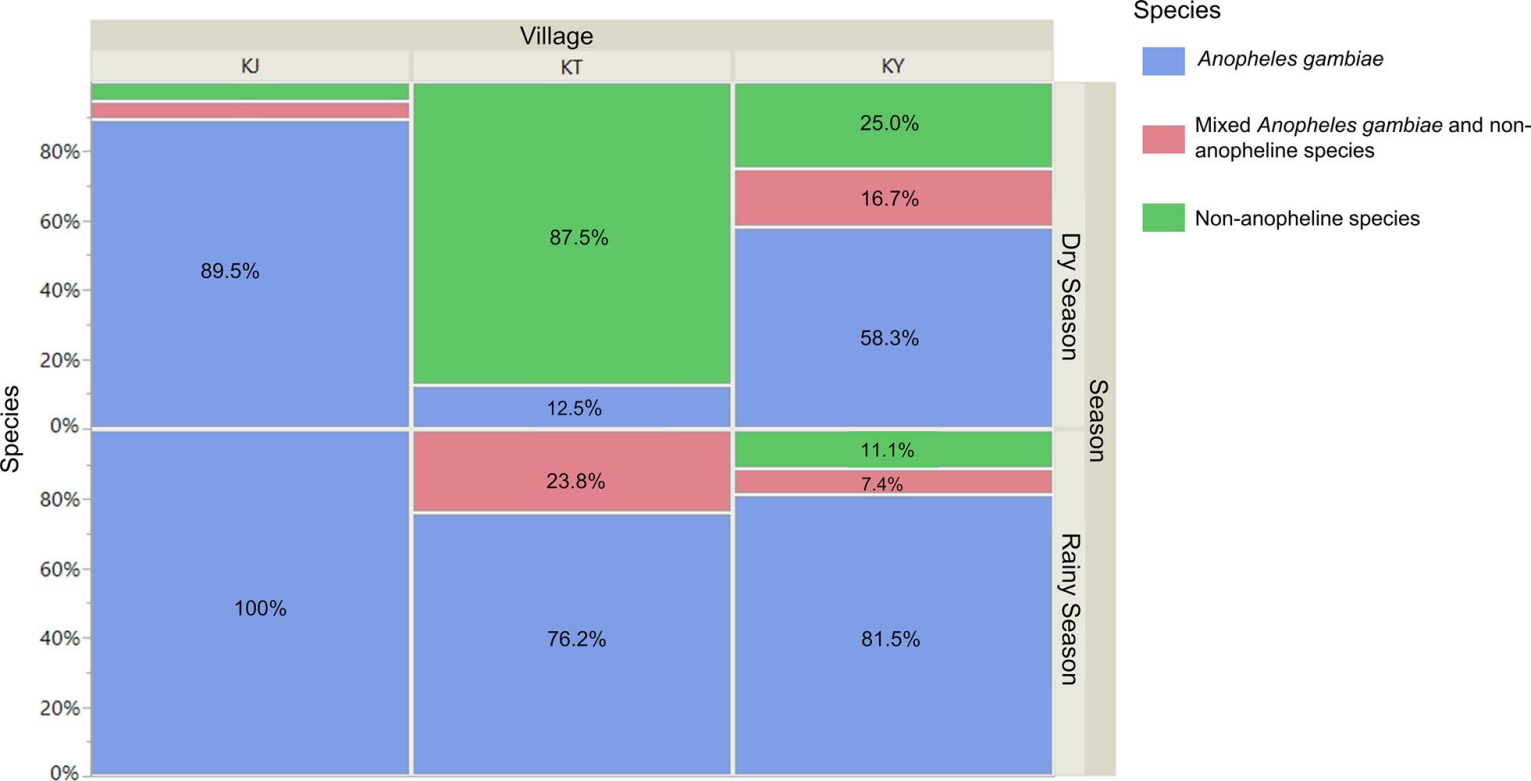
Percentage swarm species composition by season across the three villages

Most (> 70 percent) of swarms sampled were monospecific, solely composed of *An. gambiae* s.s. mosquitoes (Fig. 8). However, some mixed genus swarms containing both *An. gambiae* s.s. and *Culex* mosquitoes were also observed. Swarms composed of purely non-anopheline species were also present, including those of *Aedes*, *Culex*, mixed *Aedes* and *Culex*, and *Mansonia*. The proportion of *An. gambiae*, non-anopheline, and mixed species swarms varied significantly between villages (Poisson distribution general linear model: *df* = 2, likelihood ratio LR = 25.9, *P* < 0.001) and seasons of sampling (*df* = 1, likelihood ratio LR = 13.8, *P* < 0.001). A significantly larger proportion of non-anopheline and mixed species swarms were sampled in the village of Katuuso and in the dry season compared to the rainy season (Fig. 8).

### Swarm marker preference

**Fig. 9:**
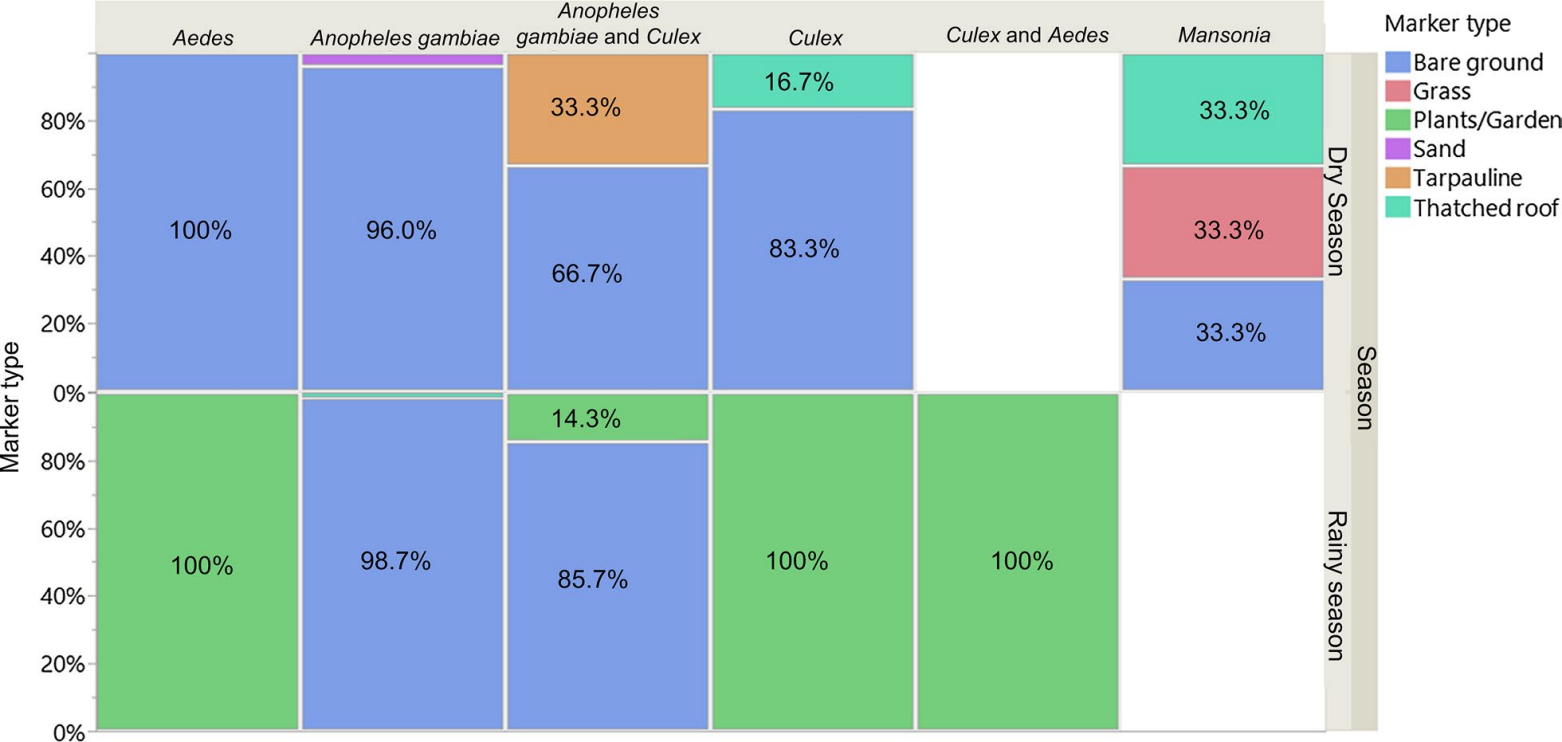
Percentage swarm marker preferences by species and season

Mosquito swarms were sampled over a range of different markers including bare ground, tarpaulin, sand heaps, thatched roofs, and grass (Fig. 9). Analysis showed that mosquito swarms composed of purely *An. gambiae* s.s were significantly associated with bare ground markers regardless of season ((χ^2^ = 15.7, *P* < 0.001), while mixed species swarms regardless of whether or not they contained *An. gambiae*, and non-anopheline swarms were not significantly associated with any particular markers (*P* > 0.005) (Table 1). No significant variation in swarm marker preference was found between villages.

**Table 1:** Parameter estimates of the binomial generalised linear model used to identify the effects of swarm species composition, season, and village on swarm marker preference

Term	L-R chi square	*P* value
Intercept	0.0006753	0.9793
Species [*Aedes*]	0.1215544	0.7274
Species [*An. gambiae*]	15.677849	< .0001*
Species [*An. gambiae and Culex*]	1.3085113	0.2527
Species [*Culex*]	0.3749695	0.5403
Species [*Culex and Aedes*]	1.7315897	0.1882
Season [dry Season]	0.0835542	0.7725
Village [KJ]	1.1596446	0.2815
Village [KT]	0.0696336	0.7919

*Significant difference

### Swarm height above ground

**Fig. 10:**
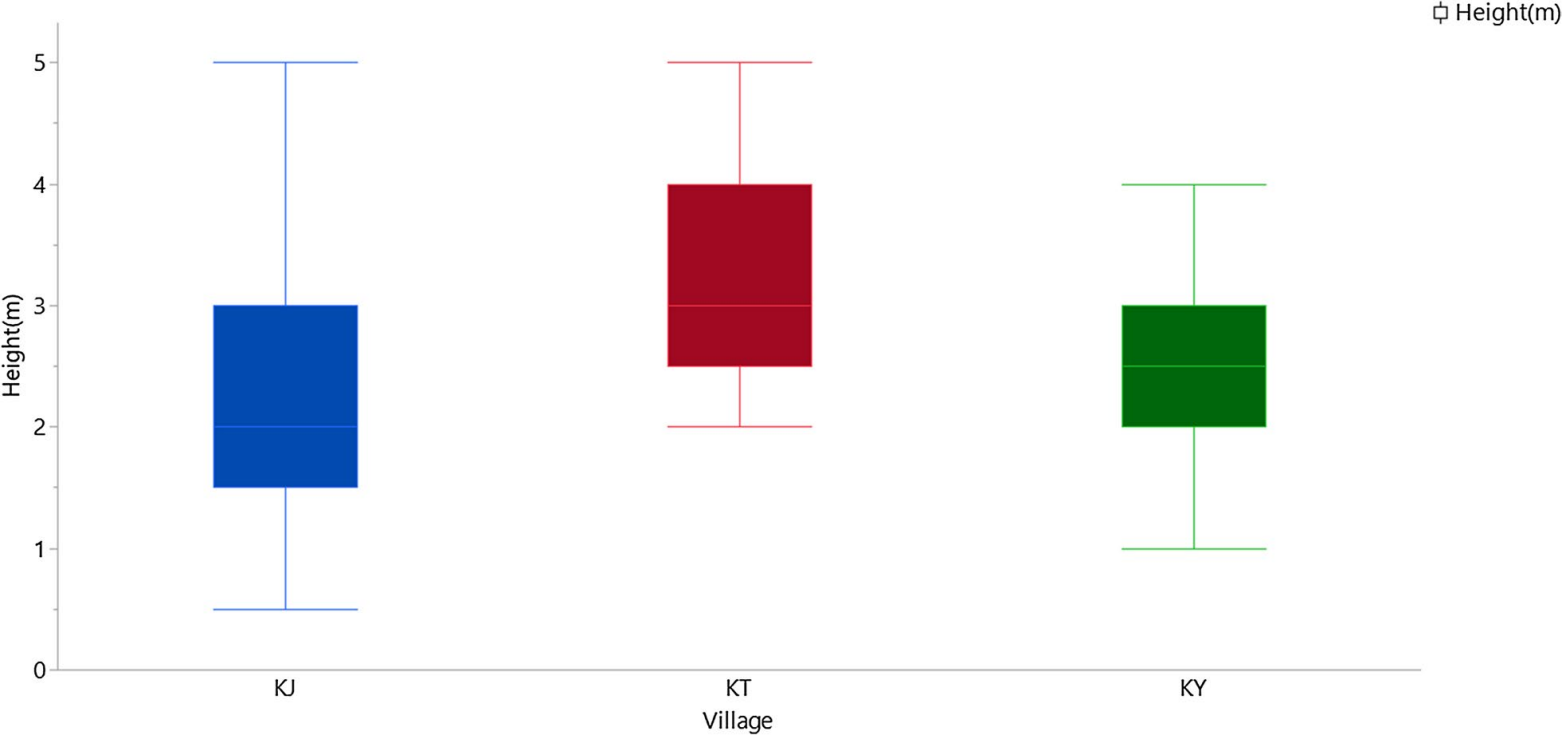
Median swarm height by village

Swarm height varied significantly between villages but did not vary significantly by season (Wilcoxon test: *df* = 1, χ^2^ = 0.89, *P* = 0.345) (Fig. 10). Swarms were found closest to the ground in Kayonjo village (median = 2.16 m ** 0.88SD) and highest from the ground in Katuuso village (median = 3.13 m ** 0.85 SD).

## Discussion

Swarm sampling has been shown to be an effective male mosquito sampling method in West Africa [8, 23] but has presented more challenges in East Africa. Several factors contribute to this: swarms have been harder to find and, as seen in this study, can be high off the ground. Except for a handful of studies published over nearly 40 years and based in Tanzania, no studies had been published on *An. gambiae* s.l mosquito swarms in East Africa [3, 9, 20]. This study has for the first time to our knowledge characterised *An. gambiae* s.l mosquito swarms in Uganda and demonstrated swarm sampling as an effective mosquito collection method in rural Ugandan villages.

During the rainy seasons, sweep netting of swarms was highly effective and over a thousand male mosquitoes were caught during the 2 years of sampling. In the dry seasons, however, fewer swarms were available to sample and only a few hundred males were captured during the same time period. Similar to West African studies of mosquito swarms, this study found that mosquito swarm densities peaked during the rainy season [8]. These results suggest that disruptions caused by rainfall do not reduce swarm densities during the rainy seasons, which is therefore the best time to sample swarms of *An. gambiae* s.l in Uganda when swarms are generally larger and thus easier to spot than those observed during the dry seasons.

Swarms were observed to form in inhabited areas close to the village houses, where mated females are more likely to find blood meals. This is similar to previous studies which have found that the major malaria vectors *An. funestus* and *An. gambiae* s.l clustered around village households [7, 9, 11, 16]. Although swarm samplers surveyed all areas of the village, no swarms were found near the larval habitats which are mostly located in gardens on the village outskirts 0.5–1.5 km from the homesteads. This suggests substantial local mobility by females seeking suitable oviposition sites. The restriction of mosquito swarms in a village close to household locations could prove useful when targeting swarms for mosquito control intervention, as well as for identifying the major malaria vector species in a given location. A study analysing male mosquito collection methods in Uganda done in 2016 showed a correlation between the size of swarms sampled and the density of male mosquitoes collected in eaves of adjacent houses, with most swarms forming next to homesteads like we have observed here [16].

*Anopheles gambiae* s.s was mainly found in monospecific swarms, which was expected as these localities are *An. gambiae* strongholds with hardly any other *Anopheles* species present. However, a few mixed genus swarms containing *Culex* spp. mosquitoes were also observed. Other studies have demonstrated mixed species swarms before. In Zambia, a mixed swarm of *Anopheles funestus* and *An. leesoni* was found [24]; in Tanzania, mixed swarms of *An. arabiensis* and *An. funestus* have been observed [12]; in West Africa, mixed swarms of *An. coluzzii* and *An. gambiae* s.s. have also been reported [8]. However, this is the first instance, to our knowledge, of mixed genus swarms, including of *Culex* and *An. gambiae* s.s mosquitoes caught in the same sweep net. Data collected from human landing catch collections (HLC) have shown hundreds of *Culex* spp. mosquitoes being collected in all three of these villages over this time period (unpublished data). Since the dry season leads to the desiccation of vegetation within the villages creating bare ground where grass may have previously been, this may have resulted in a large proportion of both *Culex* and *Anopheles* swarming over bare ground. This combined with the generally high *Culex* spp. mosquito densities in our study area could be contributing to the occurrence of these mixed genus swarms. In a previous study, we found Katuuso to have the highest *Culex* densities with HLC in the dry season, collecting 10 times as many *Culex* mosquitoes compared to *An. gambiae* s.l. This could possibly be leading to competition between the species for swarm markers and explain the higher percentage of mixed genus swarms found in in Katuuso village (Fig. 8).

An ecological separation in swarm marker use was evident as most (> 98%) of exclusively *An. gambiae* s.s swarms recorded during this study were located over bare ground markers in all three villages sampled. Statistically, only *An. gambiae* s.s swarms demonstrated a significant preference for a specific marker (bare ground) regardless of season. Swarms located above other contrasting materials like black tarpaulin and grass were all found to be non-anopheline. This contrasts with studies in West Africa and Tanzania which have found multiple *Anopheles* spp. including *An. gambiae* s.l swarms over a variety of markers [11, 23]. This is likely due to differences in the local mosquito population species as these studies included anopheline species other than *An. gambiae* s.s. However, detailed studies in Burkina Faso found that *An. gambiae* s.s. preferred to swarm over bare ground, unlike *An. coluzzii*, which favoured darker objects, woodpiles, and places such as wells [15–18, 18–21, 23]. Interestingly, bare ground was also the most common swarm marker for *An. gambiae* s.s. in Tanzania [10]. This species-specific swarm marker preference is thought to prevent the occurrence of mixed swarms with other species of the *An. gambiae* s.l. complex and thus to act as pre-mating barrier to hybridization [15] and possibly to reduce competition for swarm markers between different mosquito species.

The mean swarm height varied significantly between villages, unlike observations of *An. arabiensis* and *An. funestus* in Tanzania [11, 12]. However, the range of median swarm heights (Kayonjo village 2.16 m, Katuuso village 3.13 m, and Kibbuye village 2.61 m) remained similar to those found in West African studies of *An. gambiae* s.l swarms [18, 25]. Swarms in Katuuso village were sometimes found at heights of 4 m. Previous studies in Burkina Faso have indicated *An. coluzzii* swarms that would rise up to this height if a view of the horizon was obstructed [9]. This could be evidence of *An. gambiae* s.s exhibiting similar behaviour. These heights were above what samplers could comfortably reach. Therefore, new swarm sampling techniques targeting very high swarms may need to be explored in future studies. Evidence of similarities in swarm behaviour between similar species in West and East Africa is important for the development of vector control tools targeting mosquito swarms as it suggests that these could be successful across a wide range of locations.

## Conclusions

*Anopheles gambiae* s.l. swarms can be effectively located and sampled in South Central Uganda. *Anopheles gambiae* swarm behaviour varies seasonally and by location in terms of size and abundance but remains similar in terms of swarm marker preference (bare ground) and proximity to households across seasons and locations. These are therefore useful parameters for *An. gambiae* swarm location in these areas. Tools are needed that allow swarm capture at up to 5 m above the ground as many swarms were quite high off the ground. While mixed species swarms have been reported before, this is the first documented instance of mixed genus swarms found in Uganda, which warrants further study to fully understand the species mating dynamics. The characterization of the mating behaviour of *An. gambiae* mosquito species in Uganda in terms of swarm species composition, height above ground, swarm sizes, swarm marker preference, and swarm distribution done in this study will enable researchers to reliably find and sample these swarms since we have successfully demonstrated the ability to reproducibly sample and analyse swarms of *An. gambiae* s.s mosquitoes in rural Ugandan villages. Despite challenges encountered in the height above ground of some swarms, this study has provided ecological information on swarm occurrence, and the data obtained on *An. gambiae* s.s species mating behaviour in this study will support further study of *An. gambiae* mosquito swarms and consequently the development of novel mosquito vector control tools that rely on understanding of this behaviour.

### Supplementary Information

**Additional file 1:** Data file.

## Data Availability

The data pertaining to this manuscript has been made available as part of its Additional file 1.

## Abbreviations

ITN: Insecticide-treated net
IRS: Indoor residual spraying
ACT: Artemisinin-based combination therapy
PCR: Polymerase chain reaction
